# The origin of YouTube videos on hereditary angioedema matters

**DOI:** 10.1186/s13223-025-00947-6

**Published:** 2025-03-19

**Authors:** Pelin Korkmaz, Ilkim Deniz Toprak, Zeynep Kilinc, Derya Unal, Semra Demir, Asli Gelincik

**Affiliations:** Faculty of Medicine, Department of Internal Medicine, Division of Allergy and Clinical Immunology, Istanbul University, Istanbul, Turkey

## Abstract

**Background:**

Hereditary angioedema (HAE) is a rare, potentially life-threatening condition that requires accessible and reliable information. YouTube has emerged as a significant source of health-related content, offering valuable insights while posing the risk of misinformation that warrants caution among users. The aim of this study was to evaluate the popularity, reliability, understandability, actionability, and overall quality of YouTube videos related to HAE.

**Method:**

A search was conducted on YouTube using the term “hereditary angioedema.” Videos were categorized based on their origin (health or nonhealth) and content type (medical professional education (MPE), patient education (PE), patient experience, or awareness). The quality, reliability, understandability, and actionability of the videos were assessed via the Global Quality Scale (GQS), the Patient Education Materials Assessment Tool for Audiovisual Materials (PEMAT-A/V), and the Quality Criteria for Consumer Health Information (DISCERN) tool. Three independent allergists evaluated the videos.

**Results:**

Out of 135 reviewed videos, 111 met the inclusion criteria. The health group presented significantly higher scores than did the nonhealth group in several metrics: PEMAT-A/V understandability (83, IQR: 56–92, p = 0.001), total DISCERN score (37, IQR: 3–45, p < 0.001), reliability (23, IQR: 19–26, p < 0.001), treatment (15, IQR: 8–21, p = 0.007), and modified DISCERN score (3, IQR: 2–4, p = 0.002). Health videos were uploaded more recently (p = 0.006), while awareness videos tended to be older than more recent MPE videos (p = 0.002). The MPE videos had the longest duration, whereas the awareness videos had the shortest duration (p < 0.001). Video quality scores, assessed via the GQS, were higher in both the MPE and PE groups (scores: 3, 4, and 5; p = 0.005). Compared with the other groups, the MPE group also had significantly higher PEMAT-A/V understandability scores (91, IQR: 70.75–92, p < 0.001), total DISCERN scores (40, IQR: 30.75–49.5, p < 0.001), reliability scores (24, IQR: 21–27.25, p < 0.001), and overall scores for moderate to high quality (83, 74.8%, p = 0.002).

**Conclusion:**

YouTube videos on HAE uploaded by health care professionals generally offer higher-quality information, but their overall reliability remains suboptimal. There is a pressing need for higher-quality, trustworthy content, particularly from professional medical organizations, to address this gap.

**Supplementary Information:**

The online version contains supplementary material available at 10.1186/s13223-025-00947-6.

## Introduction

Hereditary angioedema (HAE) is characterized by recurrent episodes of cutaneous or submucosal oedema that can affect the extremities, face, gastrointestinal tract, genitals, and larynx [1, 2]. The most common cause of HAE is a deficiency or dysfunction of C1 esterase inhibitor (C1-INH), leading to excessive bradykinin production. In addition to C1-INH-deficient HAE, there is also a form of HAE with normal plasma levels and C1-INH function. This form encompasses a heterogeneous group of patients, some with one of eight documented mutations and others with a family history of angioedema without identifiable mutations (HAE-nl-C1INH) [3–10]. The 2021 guidelines from the World Allergy Organization (WAO) and the European Academy of Allergy and Clinical Immunology (EAACI) suggest considering an HAE diagnosis in patients with recurrent skin swelling, gastrointestinal pain, or laryngeal oedema [3]. The diagnosis of C1-INH-deficient HAE requires the measurement of serum C1-INH and C4 levels, along with the assessment of C1-INH function [11]. For HAE-nl-C1INH patients, diagnosis relies on identifying relevant mutations or a family history of HAE in conjunction with clinical findings. However, misdiagnosis and delays in diagnosis are common in practice [12, 13].

The comprehensive classification of HAE can also pose diagnostic challenges. Additionally, access to appropriate treatment remains a significant issue for patients and their families, particularly in countries where diagnostic tests for HAE are costly or unavailable. Consequently, delays in diagnosis or misdiagnosis are common [14]. This complexity is magnified by the diverse classifications of HAE, presenting significant obstacles to timely and accurate diagnosis. The scarcity of specialized information on rare diseases on social media heightens the risk for HAE patients to encounter unverified or misleading content. Therefore, raising awareness of this rare disease among health care providers and the public is essential.

The effective management of HAE is crucial not only for physical health but also for improving patients' quality of life (QoL). HAE is a rare disorder marked by unpredictable acute attacks that significantly affect patients' QoL, affecting aspects such as psychiatric health, social activities, travel, and work or school, and potentially leading to hospitalization. The unpredictable nature of these attacks presents challenges not only for patients but also for their families and employers [15]. Many individuals with HAE also experience significant psychological challenges, such as anxiety and depression, which compound the physical burden of the disease [16]. Furthermore, the high mortality rate among undiagnosed patients highlights the critical need for increased awareness among health care providers to prevent misdiagnosis and ensure timely treatment [17]. Many patients experience considerable diagnostic delays, with studies showing that the average time to diagnosis can exceed 10 years [18]. Additionally, the rarity of HAE may lead health care professionals to overlook the condition, resulting in missed or delayed diagnoses. Consequently, patients who receive inaccurate diagnoses may turn to social media platforms for information, where they often encounter misinformation; this creates a vicious cycle, as the lack of accurate information further complicates their understanding and management of the disease. Given the critical role of accurate information in managing HAE, particularly in the age of social media, it is essential to explore how platforms such as YouTube can serve as both a source of support and misinformation for patients. Therefore, the results obtained in this study emphasize the public health relevance of HAE and the unique challenges faced by patients on social media platforms, ultimately highlighting the necessity for credible resources in the management of this disorder.

With the growing availability of the internet, individuals now have easy access to a vast array of medical information online [19]. Through social media platforms, health care professionals and patients can share and exchange diverse information and personal experiences. As an open platform, YouTube enables the unrestricted uploading of videos on virtually any topic. It is estimated that over 2 billion individuals access YouTube each month [20]. Currently, as the most widely used online media-sharing platform worldwide, YouTube plays a pivotal role in disseminating medical information, with numerous studies underscoring its value as a source of health-related content [19, 21–23]. However, a significant concern is the lack of policies to verify video creators' credibility and qualifications, which allows unreviewed content to be uploaded freely [24]. This lack of quality control presents risks for patients seeking reliable information and may mislead medical professionals, students, and family members. Recent research underscores these concerns. For example, a study reported that the quality of YouTube videos recommending exercises during the COVID-19 lockdown was generally low and did not align with WHO recommendations. This study highlighted the need for effective tools and strategies to help users identify reliable content and filter out inaccurate or low-quality videos [25]. Another study evaluated the quality of YouTube videos on incontinence information following cancer surgery and reported strong correlations among quality assessment scales, which supported their effectiveness. The study also recommended policy improvements and tools to help patients access reliable health content [26]. Studies on various medical topics suggest substantial variability in the quality and reliability of health-related content on YouTube, with some videos potentially providing incomplete or inaccurate information. As highlighted by various studies, YouTube serves as a vast repository where misinformation and disinformation can circulate freely, posing significant risks of confusion and the spread of inaccurate information [27]. The issue of misinformation underscores the need for careful evaluation and strategies to improve the accuracy and credibility of health information on YouTube. By acknowledging the interdisciplinary nature of YouTube as a platform for health information and leveraging evidence-based practices, health care providers can enhance digital health literacy, empower individuals to make informed decisions regarding their care, and transform YouTube into a valuable resource for sharing and disseminating reliable health-related information [28]. In general, videos uploaded by health care institutions or professionals tend to demonstrate greater reliability and quality than those created by nonexperts. However, to the best of our knowledge, no systematic studies have specifically examined YouTube content related to HAE.

This study aims to fill this gap by evaluating YouTube's role as a communication tool for HAE information. In line with findings from other medical fields, we hypothesize that videos uploaded by health care institutions or professionals will exhibit higher reliability and quality compared to those created by nonexpert individuals. Specifically, our objectives are to (1) assess the popularity, reliability, understandability, actionability, and overall quality of HAE-related videos; (2) compare the sources and purposes of these videos; and (3) identify the most reliable sources of HAE information available on YouTube. Accurate information is crucial for undiagnosed HAE patients who may experience life-threatening attacks, highlighting the public health importance of this study. By addressing these aims, we seek to provide a comprehensive understanding of YouTube's role in disseminating HAE information and to guide both patients and health care professionals in evaluating the quality of online resources.

## Materials and methods

### Study design

In this study, we conducted a search on the YouTube platform (https://www.youtube.com) using the keywords “hereditary angioedema” in June 2023, following the ethical principles outlined in the Helsinki Declaration. To minimize search history and cookie bias, we cleared the browser history and used incognito mode with default search settings, aiming to replicate typical user behaviour. Additionally, to prevent bias related to the country or content creator, we disabled location services and set English as the default language. We aimed to replicate a simple search strategy that could be conducted by any person without using filters. Therefore, YouTube’s ranking algorithm was used to sort video results by relevance. Unlike some studies that limit their selection to the first 50 or 150 videos, we included all videos accessible through the YouTube platform at the time of the search. This approach was selected to maximize the comprehensiveness of our dataset and reflect the full range of available content on this topic. To maintain consistency, new videos uploaded during the screening process were excluded from the dataset. The 135 videos available to viewers were selected based on their relevance according to YouTube’s sorting algorithm at that specific time. As of June 2023, the oldest video was uploaded 153 months earlier, whereas the most recent video was uploaded just 1 month prior.

The identified videos were added to a spreadsheet, and their URLs were used for subsequent screening and coding. In the initial screening phase, three researchers independently reviewed the videos to apply the inclusion and exclusion criteria. For videos where discrepancies arose regarding the criteria, the researchers convened to discuss and reevaluate these videos collaboratively, ensuring consensus in the final decision. Three independent allergists, each with relevant experience, evaluated the videos in separate environments over a 4-week period to ensure unbiased analysis. An initial set of 135 videos was screened, and a flow diagram was created in accordance with PRISMA guidelines, illustrating the videos that were excluded based on the specified criteria [29, 30]. This manual process was chosen to ensure a thorough evaluation of each video's compliance with the criteria.

**Fig. 1:**
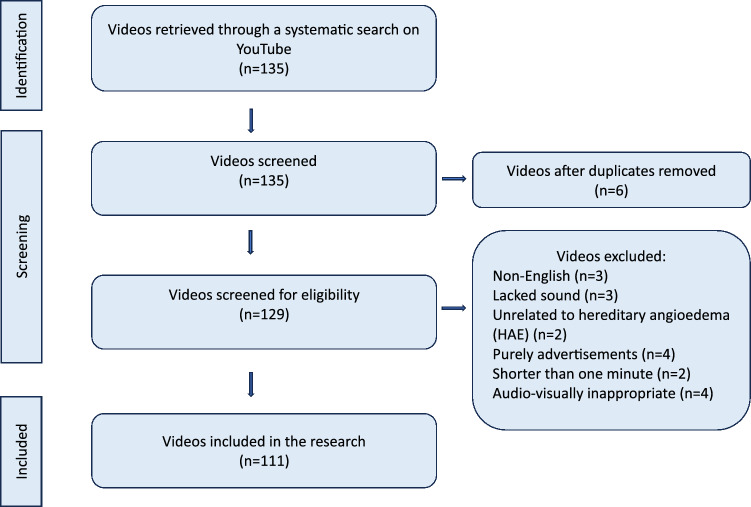
Flow diagram of video selection process

The exclusion criteria were as follows: videos that were non-English, lacked sound, were unrelated to HAE, were purely advertisements, were shorter than one minute, were audiovisually inappropriate, or were duplicates. The inclusion criteria were used to select videos that were in English, had sound, were related to HAE, were not purely advertisements, were at least one minute in length, were audiovisually appropriate, and were not duplicates. From an initial set of 135 videos, a detailed screening process was applied, with each video evaluated individually by three independent allergists in separate settings. Videos were excluded if they were non-English (n = 3), lacked sound (n = 3), were unrelated to hereditary angioedema (HAE) (n = 2), were purely advertisements (n = 4), were shorter than one minute (n = 2), were audio-visually inappropriate (n = 4), or were duplicates (n = 6). This process resulted in a final selection of 111 videos, each reviewed independently to ensure an unbiased analysis (Fig. 1).

### Evaluation and categorization of the videos

All YouTube videos were evaluated via assessment tools for general information, quality, reliability, understandability, and actionability. The videos were then compared across two origin categories and four content-purpose subgroups. The videos were categorized into two groups based on the presenter and/or YouTube channel: the 'Health Group' and the 'Nonhealth Group', following the methodology used in previous studies [31, 32]. A video was classified as part of the health group if the presenter was a medical doctor, paramedic, nurse, pharmacist, or an unspecified health care professional. Similarly, if the channel belonged to a medical doctor, paramedic, nurse, pharmacist, health care facility, training or educational centre/company, nonprofit medical association, or governmental medical organization, the video was also considered part of the health group. All other presenters and channels were classified as part of the nonhealth group.

In addition to categorizing the videos by their origin, all videos were further subdivided into four subgroups based on their content and purpose:

• Medical Professional Education (MPE): These videos are aimed primarily at health care professionals and focus on providing advanced knowledge and skills related to HAE.

• Patient Education (PE): These videos are designed to educate the general public about HAE, offering important information to enhance the understanding and management of the condition.

• Patient Experience: Videos in this category centre on the personal experiences of patients or their relatives, who share individual narratives without an educational component.

• Awareness: These videos focus solely on raising awareness about HAE without offering educational content or sharing personal experiences.

#### Evaluation of general information

General information included data on views, likes, time of upload (in months), and duration (in minutes) of the YouTube videos. To provide a deeper understanding of user engagement, we calculated the view-to-month ratio to assess video popularity.

#### Assessment of quality, reliability, understandability and actionability

The quality, reliability, understandability and actionability of the videos were assessed via several tools: the Global Quality Scale (GQS) [27], the Patient Education Materials Assessment Tool for Audiovisual Materials (PEMAT-A/V) [33], the Quality Criteria for Consumer Health Information (DISCERN) [34], and a modified version of DISCERN [35] (Supplemental Table 1). Video quality and streaming were evaluated via a 5-question GQS score, where a higher GQS score indicated superior content quality and information. To evaluate the understandability and actionability of the videos, the PEMAT-A/V score was applied. PEMAT assesses educational material in two ways: understandability, which enables individuals with varying levels of health literacy to comprehend and identify key video content, and actionability, which determines whether viewers can take appropriate actions based on the materials presented. The DISCERN scoring system, developed by Charnock et al. in 1999, consists of 16 questions designed to evaluate the quality of information. Each question is scored on a scale from 1 to 5 points. The questions are categorized into three sections: reliability (questions 1 to 8), quality of information about treatment options (questions 9 to 15), and an overall score (question 16). DISCERN scores are categorized as follows: excellent is denoted by scores of 63–75 points, good is denoted by scores of 51–62 points, fair is denoted by scores of 39–50 points, poor is denoted by scores of 27–38 points, and very poor is denoted by scores of 16–26 points. In 2012, Singh et al. employed a modified version consisting of 5 questions. According to this scoring system, each question is awarded 1 point for a 'yes' answer and 0 points for a 'no' answer, with each video receiving a total score ranging from 0 to 5 points. Each of these scoring systems was rated on a scale, with higher scores indicating greater reliability. To ensure objectivity, the average of three independent results was used.

#### Evaluation of specific information on hereditary angioedema

The presence of detailed information about HAE was examined in each video. Information on family history, genetic inheritance, symptoms, response to antihistamines, prodromal symptoms, and the frequency and triggers of attacks was analysed. Additionally, all videos were categorized based on whether they contained laboratory findings such as increased serum bradykinin levels, low serum levels of C1-INH and C4, and low C1-INH function. Videos were also evaluated for information on the possibility of delayed diagnosis or misdiagnosis of the disease. Information on therapeutic interventions, including bradykinin receptor antagonists, C1-INH extracts, kallikrein enzyme inhibitors, androgens, tranexamic acid, berotralstat, and lanadelumab, was analysed.

## Statistical analysis

The data were analysed via the Statistical Package for Social Sciences (SPSS). Continuous variables, such as views, likes, upload time (in months), video duration (in minutes), the views per month ratio, the likes per month ratio, and the likes-to-views ratio, were tested for normality via the Kolmogorov‒Smirnov test, with p values < 0.05 indicating a nonnormal distribution. Given that the data did not follow a normal distribution, nonparametric tests were applied for statistical comparisons.

For the statistical analysis, the Kruskal‒Wallis test was used to compare continuous variables across more than two categorical groups (awareness, patient experience, PE, and MPE). The continuous variables analysed in relation to these categories included views, likes, duration, upload time, views per month, PEMAT A/V actionability, PEMAT A/V understandability, DISCERN total score, reliability, treatment information, and modified DISCERN score. For variables found to have statistically significant differences between groups, post hoc pairwise comparisons were conducted via the Dunn–Bonferroni correction to identify which specific groups differed from each other.

For comparisons between two categorical groups (i.e., health vs. nonhealth videos), the Mann‒Whitney U test was applied to assess differences in continuous variables, including views, likes, duration, upload time, views per month, PEMAT A/V actionability, PEMAT A/V understandability, DISCERN total score, reliability, treatment information, and modified DISCERN score. These nonparametric tests were chosen for their suitability in handling nonnormally distributed data and for their robustness as alternatives to parametric tests. While they allowed for the detection of significant differences between groups, the ordinal nature of these tests limits the interpretation regarding the magnitude of these differences.

For categorical comparisons, the overall DISCERN and GQS scores were analysed by categorizing scores of 3 and above as moderate-to-high quality, whereas scores below 3 were deemed poor quality [36]. For comparisons between the scores (overall DISCERN and GQS) and the categorical health and nonhealth groups, we used the chi-square test. Additionally, the Spearman rank correlation test was employed to examine the relationships between the overall DISCERN and GQS scores and the categorical variables of awareness, MPE, PE, and patient experience. These tests facilitated the analysis of associations between categorical quality metrics and other categorical groupings within the study.

We presented scores for continuous variables, such as PEMAT-A/V actionability, PEMAT-A/V understandability, DISCERN reliability, DISCERN treatment, and modified DISCERN, as median values with interquartile ranges (IQRs 25–75). The p values for these scores were calculated via the Kruskal‒Wallis and Mann‒Whitney U tests as appropriate.

To enhance the reliability analysis, the interclass correlation coefficient (ICC) was calculated for GQS, PEMAT-A/V actionability, PEMAT-A/V understandability, DISCERN reliability, overall DISCERN, DISCERN treatment, and modified DISCERN scores. We also included 95% confidence intervals (CIs) in the analysis to improve result interpretability. Reliability was classified as follows: values < 0.5 indicated poor reliability, values between 0.5 and 0.75 indicated moderate reliability, values between 0.75 and 0.90 indicated good reliability, and values > 0.90 indicated excellent reliability [37]. ICC values greater than 0.75 were considered indicative of good correlation. Statistical significance was set at p < 0.05 throughout the analysis.

## Results

### Overview of video data

A total of 135 video URLs were reviewed by three blinded allergists, leading to 111 videos that met the inclusion criteria. These videos collectively comprised 34 h and 39 min of content, accumulating a substantial 380,951 views and 4,815 likes. The median video duration was 5 min, with a median upload time of 38 months. Notably, the median number of views per video was 371, indicating substantial viewer engagement. The engagement metrics revealed a median of 16 views per month, underscoring the popularity of these videos as valuable sources of health information. No significant difference in video popularity was observed between the health and nonhealth groups. The health group videos were significantly more recent, with a median upload time of 34 months, than the nonhealth group videos, with a median upload time of 71 months (p = 0.006). However, no significant differences were observed between the health and nonhealth groups in terms of views, likes, durations, or views-to-months ratios (Table 1).

**Table 1:** Assessment of popularity, reliability, understandability, actionability, and general characteristics of video content in health and non-health groups

	Total (n = 111)			Health (n = 95)			Non-health (n = 16)			p
	Median	Min	Max	Median	Min	Max	Median	Min	Max	
Views (n)	371	104	2665	328	83	2665	1091	267	3106	NS
Likes (n)	5	1	43	5	1	36	7.5	4	51	NS
Duration (in minutes)	5	2	19	6	2	23	4.5	1	16	NS
Uploading time (in months)	38	16	72	34	16	68	71	55	98	0.006
Views/Months ratio	16	2	51	17	2	51	13	4	56	NS
PEMAT-A/V-actionability score (%)	0	0	0	0	0	0	0	0	0	NS
PEMAT-A/V-understandability score (%)	78	56	92	83	56	92	56	41	70	0.001
DISCERN-total score	35	27	44	37	3	45	24	21	32	p < 0.001
DISCERN-reliability score	22	16	26	23	19	26	15	11	19	p < 0.001
DISCERN-treatment score	14	7	20	15	8	21	7	7	14	0.007
Modified DISCERN score	3	2	3	3	2	4	2	1	3	0.002

*NS* Not significant, *PEMAT-A/V* Patient Education Materials Assessment Tool for Audio-visual (0–100, higher scores indicate better understandability and actionability). DISCERN – A tool for assessing the quality of written health information on treatment choices (16–75, higher scores indicate better quality. Modified DISCERN – An adaptation of the DISCERN tool for audio-visual materials (0–5, higher scores indicate better quality)

### Video categorization and upload trends

Among the videos, 95 (85.5%) were health-related, whereas 16 were non-health-related (Table 1). Health-related videos were uploaded primarily by medical doctors (58%), followed by health advocates (31.6%), allied health personnel (8%), and paramedics/nurses (2.4%). Notably, nonprofit medical associations uploaded the largest share of videos (38.9%), closely followed by training channels (37.9%). Smaller contributions came from health care facilities (12.6%) and medical doctor channels (5.3%). Unclassified and governmental medical organization channels accounted for 4.2% and 1.1% of uploads, respectively. In contrast, the nonhealth group consisted solely of videos uploaded by patients or their relatives.

The health group content focused primarily on MPE (64.2%), with less emphasis on awareness (24.2%), patient experiences (14.4%), and PE (5.3%). In contrast, nonhealth videos mainly featured patient experiences (62.5%), with smaller portions dedicated to awareness (25%), PE (6.3%), and MPE (6.3%).

Among the video types, MPE videos were the most common, comprising 56% of the total. The median duration for MPE videos was significantly longer at 11 min (5–42) than for other types (p < 0.001), and post hoc analysis revealed that this difference was primarily between the MPE and awareness groups (p = 0.002). Compared with the other types, the MPE videos were also the most recent, with a median upload time of 27 months (13.5–53.25) (p = 0.002), and post hoc analysis confirmed a significant difference between the MPE and awareness groups (p < 0.001). Awareness videos had the shortest median duration at 2 min (1–4) (Table 3). However, no significant differences were observed between the four video types (MPE, PE, patient experiences, and awareness) in terms of views, likes, or views-to-months ratios.

### Assessment of quality, reliability, understandability, and actionability results among categories and subgroups

The quality, reliability, understandability, and actionability of the videos were assessed via several tools: the GQS, the PEMAT-A/V, the DISCERN, and a modified version of DISCERN. Video quality and streaming were evaluated via a 5-question GQS score, with a higher GQS score indicating superior content quality and information. Among all videos, 62 (55.9%) scored 3 or above on the GQS, suggesting higher quality in a substantial portion of the content. No significant difference was observed between the health and nonhealth groups in terms of the number of poor-quality or moderate-to-high-quality videos (Table 2).

**Table 2:** Assessment GQS and DISCERN-overall scores of video content in health and non-health groups

	Total (n = 111)		Health (n = 95)		Non-health (n = 16)		p
	Poor quality n (%)	Moderate-high quality n (%)	Poor quality n (%)	Moderate-high quality n (%)	Poor quality n (%)	Moderate-high quality n (%)	
GQS	49 (44.1)	62 (55.9)	42 (44.2)	53 (55.8)	7 (43.7)	9 (56.3)	NS
DISCERN-overall score	28 (25.2)	83 (74.8)	22 (23.2)	73 (76.8)	6 (37.5)	10 (62.5)	NS

*NS* Not significant, *GQS* Global Quality Score (1–5). DISCERN – A tool for assessing the quality of written health information on treatment choices (16–75, higher scores indicate better quality)

To evaluate the videos' understandability and actionability, we applied the PEMAT-A/V score. The median PEMAT-A/V understandability score was 78 (56–92), indicating that most videos were accessible to a broad audience. The health group had a significantly greater median score (83) than did the nonhealth group (56) (p = 0.001). In our analysis, the PEMAT-A/V actionability scores were not significantly different (NS) for the HAE YouTube videos. This result suggests that the PEMAT-A/V tool may not be adequate for assessing the actionability of HAE-related content (Tables 1 and 3).

**Table 3:** Comparison of video popularity, reliability, understandability, actionability, and general characteristics based on content aim

	Medical profession education (n:62)			Patient education (n:6)			Patient experience (n:16)			Awareness (n:27)			p
	Median	Min	Max	Median	Min	Max	Median	Min	Max	Median	Min	Max	
Views (n)	273	76	3347	1607	183	2428	821	243	3070	345	125	2751	NS
Likes (n)	5.5	1	50	10.5	1.7	59	5.5	2.2	40.7	4	1	19	NS
Duration (in minutes)	11	5	42	2.5	1	4.2	6	1.2	20.7	2	1	4	p < 0.001
Uploading time (in months)	27	13.5	53.2	64	47.7	83.7	59	21	94.7	64	25	119	0.002
Views/Months ratio	15.71	2	71.2	31	9.5	39.6	17.4	4.1	59.3	14.1	2.6	33.9	NS
PEMAT-A/V-actionability score (%)	0	0	0	0	0	0	0	0	0	0	0	0	NS
PEMAT-A/V-understandability score (%)	91	70.7	92	79	54.5	85	56	55.2	65.2	64	50	90	p < 0.001
DISCERN-total score	40	30.7	49.5	24.5	20	31	30	21.5	39.5	32	26	37	p < 0.001
DISCERN-reliability score	24	21	27.2	17.5	13	21.5	14	13	19.7	20	15	23	p < 0.001
DISCERN-treatment score	15	9.7	22	7	7	9.5	15	7	21	10	7	18	0.007
Modified DISCERN score	3	3	4	2.5	1	3	2	1	2	3	2	3	p < 0.001

*NS* Not significant, *PEMAT-A/V* Patient Education Materials Assessment Tool for Audio-visual (0–100, higher scores indicate better understandability and actionability). DISCERN – A tool for assessing the quality of written health information on treatment choices (16–75, higher scores indicate better quality. Modified DISCERN – An adaptation of the DISCERN tool for audio-visual materials (0–5, higher scores indicate better quality)

In our analysis, the health group had a median DISCERN total score of 37, corresponding to the 'poor' category, whereas the nonhealth group had a median score of 24, falling within the 'very poor' category. This difference was statistically significant (p < 0.001). Additionally, the health group had significantly higher DISCERN-reliability scores (p < 0.001), DISCERN-treatment scores (p = 0.007), and Modified DISCERN scores (p = 0.002) (Table 1). The DISCERN-overall score indicated that 83 videos (74.8%) were of moderate-to-high quality. No significant difference was observed between the health and nonhealth groups in terms of the number of poor-quality or moderate-to-high-quality videos (Table 2).

The MPE videos presented higher PEMAT-A/V understandability scores, with a median score of 91 (p < 0.001), indicating that their content is more accessible to viewers with varying levels of health literacy. Post hoc analysis revealed that the significance for PEMAT understandability was primarily between the MPE and awareness groups (p = 0.04) (Table 3).

The MPE videos also achieved significantly higher DISCERN total scores, with a median score of 40 (p < 0.001). According to the DISCERN scoring categories, the median score of the MPE videos corresponds to the 'fair' category, indicating overall moderate reliability in the evaluation of health information quality. Significant differences were observed across the DISCERN subcategories: the DISCERN-reliability score had a p value of < 0.001, the DISCERN-treatment score was significant at p = 0.007, and the Modified DISCERN score had a p value of < 0.001. Post hoc analysis revealed that the significance for both DISCERN- reliability and DISCERN total scores was primarily between that of the MPE and awareness groups (p = 0.001 and p = 0.002, respectively). For the DISCERN-treatment scores, the significance was also between that of the MPE and awareness groups (p = 0.05). Additionally, for the Modified DISCERN score, significant differences were identified between the MPE and awareness groups (p = 0.02) as well as between the patient experience and awareness groups (p = 0.02) (Table 3).

Furthermore, the GQS scores, which assess the general quality of video content, were significantly higher for educational videos, especially those classified as MPE and PE (p = 0.005) (Table 4). Additionally, MPE videos had a significantly greater number of moderate-to-high-quality videos based on the DISCERN-overall score (p = 0.002) (Table 4).

**Table 4:** Assessment GQS and DISCERN-overall scores based on content aim

	Medical profession education (n:62)		Patient education (n:6)		Patient experience (n:16)		Awareness (n:27)		p
	Poor quality n (%)	Moderate-high quality n (%)	Poor quality n (%)	Moderate-high quality n (%)	Poor quality n (%)	Moderate-high quality n (%)	Poor quality n (%)	Moderate-high quality n (%)	
GQS	21 (33.9)	41 (66.1)	2 (33.3)	4 (66.7)	8 (50)	8 (50)	18 (66.7)	9 (33.3)	0.005
DISCERN-overall score	8 (12.9)	54 (87.1)	3 (50)	3 (50)	7 (43.7)	9 (56.3)	10 (37)	17 (63)	0.002

*GQS* Global Quality Score (1–5). DISCERN – A tool for assessing the quality of written health information on treatment choices (16–75, higher scores indicate better quality)

### Analysis of the objectivity of the outcomes among the assessments of the reviewers

The ICCs were calculated for the outcome tools: 0.959 for GQS; 1 for content; 0.949 for PEMAT-A/V actionability; 0.895 for PEMAT-A/V understandability; 0.872 for DISCERN reliability; 0.839 for DISCERN overall; 0.782 for DISCERN treatment; and 0.834 for modified DISCERN.

### Review of specific information on hereditary angioedema in each video

Only 33 (30%) of the videos addressed issues of or delayed diagnosis, with no significant difference between health and nonhealth videos. The mention of specific treatments varied among the videos; 52% of health care professional videos discussed the C1-INH extract, whereas mention rates were reported for bradykinin receptor antagonists (37.8%), kallikrein enzyme inhibitors (39.8%), lanadelumab (24.5%), and tranexamic acid (19.4%). Notably, the videos covered berotralstat. Additionally, only 36% of the videos mentioned low C4 levels, 59% mentioned low C1 inhibitor levels, and 33% covered the different types of HAE.

## Discussion

The rapid proliferation of technology and the availability of diverse content on platforms such as YouTube have positioned these channels as significant sources of health-related information. Both health care professionals and patients increasingly rely on these platforms for information. However, the lack of regulatory oversight poses the risk of the dissemination of incomplete, misleading, or harmful information, which may misguide users. In our study, we found that videos created by health care professionals—particularly those aimed at education—were of higher quality than videos focused on patient experiences and awareness. However, the overall quality of health group videos has remained suboptimal. The DISCERN scores of the health group videos fell within the 'poor' category, and while the MPE videos achieved slightly higher quality, their DISCERN scores were only in the 'fair' category. These findings indicate that, despite the dominance of health care professionals in HAE-related video production, the quality of the information provided is far from ideal. This highlights the pressing need for more reliable and higher-quality content about rare diseases such as HAE on platforms such as YouTube. To better serve patients and caregivers, health care professionals must prioritize not only producing more content but also the accuracy and educational value of such content.

Our study revealed that the health group, consisting of health care professionals and institutions, uploaded the majority of HAE videos (85.5%). Within this group, medical doctors were the primary contributors (58%). These findings are consistent with studies in other medical fields, where the number of health group videos ranges from 46 to 69% [32]. Our analysis of video purpose indicated that MPE videos comprised the largest group, accounting for 56% of the total. This finding is consistent with studies in other medical fields. For example, a study by Claire et al., which examined the representation of dermatological diseases on YouTube, reported that MPE videos were the most prevalent, constituting 35% of the overall content [38]. Similarly, a study by Boyers et al. on dermatological diseases reported that 45% of the videos were MPE videos [39]. The higher rate in our study suggests that these medical conditions are uncommon, prompting health care professionals to use YouTube as a valuable source of information. This trend highlights YouTube's potential as an increasingly important educational resource, especially with the growing emphasis on educational content. With heightened awareness of HAE, it is likely that there will be an increase in videos featuring patient experiences in the future.

Our analysis demonstrated a trend towards recency in videos uploaded by health groups (including health care professionals and institutions). Videos categorized as patient experiences were the oldest, whereas those focusing on MPE were the most recently uploaded. This finding is consistent with research by Toprak et al. on adrenaline autoinjectors, which similarly identified videos uploaded by health care professionals as the most up-to-date [40]. This trend indicates that health care professionals are prioritizing the production of up-to-date and relevant educational material, thereby contributing to the enhancement of the overall quality of health-related content on YouTube; this highlights the importance of providing current and evidence-based information to improve the educational value of online health resources.

Our initial analysis revealed no overall difference in GQS between health and nonhealth videos. However, a more detailed examination by video purpose revealed that the MPE and PE videos had significantly higher GQS scores, indicating superior quality. Prior investigations assessing the quality and reliability of YouTube videos within the health domain determined that health-related videos achieved higher scores on the GQS [41, 42]. For example, a study investigating YouTube content for LAM patients revealed that videos from independent medical professionals had higher quality ratings than those from news/media sources did, with similar content distribution patterns observed [43]. However, the quality of the health group videos in our study was not overwhelmingly superior. Among all videos, 62 (55.9%) scored 3 or above on the GQS, suggesting moderate-to-high quality in a substantial portion of the content. Additionally, while the health group had a significantly higher median DISCERN score than the nonhealth group did, it still fell within the 'poor' category. The health group PEMAT actionability scores also showed only limited superiority, with a median score of 83 compared with 56 in the nonhealth group. These findings indicate that, while health care professionals and institutions contribute more frequently to HAE-related content, the overall quality of their videos remains suboptimal; this underscores the need for health care professionals to not only produce more content but also focus on enhancing the educational quality and practical applicability of such content to better serve patient needs. Furthermore, the MPE videos achieved significantly higher DISCERN total scores, which falls within the 'fair' category, indicating moderate reliability in the quality of health information. Additionally, the MPE videos had higher PEMAT-A/V understandability scores, reflecting greater accessibility for viewers with varying levels of health literacy. While these findings underscore the potential of MPE videos to deliver higher-quality and more understandable content, it is important to note that the overall performance of health-related videos remains variable; this highlights a need for further improvement in the reliability and comprehensiveness of the health information presented in these videos. The observed discrepancy, where the broader health category did not show an advantage but the MPE videos did, likely reflects the influence of the video creator expertise. Videos produced by health care professionals with domain-specific knowledge contributed to relatively better performance in these subcategories. However, this improvement does not extend to all health-related content, underscoring the variability in quality across different types of creators within the health domain. These findings emphasize the need for targeted efforts to encourage the creation of high-quality, reliable content by health care professionals. While the MPE and PE videos demonstrate the positive impact of expert involvement, broader strategies are needed to increase the overall quality of health-related videos on YouTube and address persistent gaps in reliability and accessibility.

In our study, the PEMAT actionability score was found to be inadequate for evaluating the HAE videos, as these videos lacked practical applications or visual demonstrations. Similarly, a study by Rubel et al. on sinusitis, which assessed videos via the PEMAT, revealed a shortage of high-quality online audiovisual educational material on the topic. The findings indicated that the majority of videos were both difficult to understand and lacked practical applicability [44]. However, it is noteworthy that in our study, the PEMAT understandability scores for videos in the health group and MPE group were significantly higher than those in the nonhealth group. Similarly, a study on basal cell carcinoma of the skin conducted by Steeb et al., which was assessed via PEMAT, revealed that videos created by health care professionals were significantly higher in quality than those produced by lay individuals [45]. These results suggest that while the PEMAT actionability score may not be ideal for assessing certain types of content, contributions from health care professionals generally lead to higher-quality educational materials. This finding indicates that enhancing video content by incorporating comprehensive and practical information could improve its actionability and overall educational value, particularly for rare conditions such as HAE.

During our search, we observed that videos discussing the administration of drugs used to treat HAE were inadequate. Only one video was identified, presented as a patient experience, which showed an intervention during a laryngeal attack. However, this video had poor image quality and was recorded remotely. Consequently, the available data on YouTube regarding the use of medication both during an HAE attack and for prophylaxis are insufficient. While some drug names and emergency interventions were sporadically mentioned in training videos narrated by health care professionals, comprehensive information on these topics was notably lacking. Given these limitations, we advocate for the widespread creation and dissemination of training videos that specifically address the administration of drugs for both attack management and prophylaxis.

A key limitation of our study is that YouTube continuously adds new videos, leading to a temporal limitation. Our analysis was based on videos available during the research period, meaning that newer videos, potentially containing more relevant or updated information, were not included. This temporal limitation may impact the comprehensiveness of our findings. Moreover, as YouTube content evolves rapidly, the exclusion of newer videos might result in the omission of critical insights related to HAE. Another limitation is that our study focused only on English-language videos. While English is widely spoken, a more comprehensive analysis that includes videos in other languages is necessary. Conducting multinational studies could address this limitation and improve the generalizability of our findings across diverse populations. Our study specifically used the search term " hereditary angioedema". However, since HAE is a rare condition, patients or their families might use broader terms such as "swelling" or "angioedema" when posting or searching for videos. This limitation may have caused us to miss relevant videos, further impacting the generalizability of our findings. Furthermore, considering the sample size and potential biases from the YouTube algorithm is crucial for understanding the broader implications of our study. The limited sample size of videos analysed may restrict the breadth of conclusions we can draw regarding the quality and accuracy of HAE-related information on YouTube. Additionally, since the group of individuals who may not be familiar with medical terminology is more likely to consist of patients or their families rather than health care professionals, this could have led to a higher representation of health-related videos in our analysis. This imbalance may have resulted in better outcomes for the categories of awareness and patient experience videos, which we may not have fully captured in our study, thereby affecting our overall findings.

The findings of this study have significant implications for public health, particularly as social media use expands alongside advances in technology. Platforms such as YouTube could introduce systems whereby videos containing health information are reviewed by qualified professionals prior to public access. This review process could act as a safeguard, ensuring that only verified information is made available to viewers.

Moreover, at the policy level, countries could establish dedicated divisions within their health authorities focused on social media outreach. As they do for existing educational materials on topics such as prenatal care and blood donation, these social media units could develop, review, and promote reliable health information. As many users now prefer consuming information through digital media rather than traditional print, strengthening public health content on social platforms could help individuals better understand their health conditions and recognize when to seek professional assistance. When reinforced by platform-level interventions and policy shifts, these efforts could foster a more reliable online health information environment, reaching a broader audience than individual initiatives alone can achieve.

## Conclusion

In conclusion, this study evaluated the quality of YouTube videos related to HAE, which were uploaded primarily by health care professionals, with MPE videos being the most common. Using validated tools such as GQS, PEMAT A/V, DISCERN, and Modified DISCERN, we found that while health group videos generally provide more reliable information, their quality often falls within the 'fair' or 'moderate' categories, highlighting the need for improvement. Additionally, the risk of encountering misleading or incomplete content, particularly concerning rare diseases such as HAE, persists, highlighting the need for continuous improvement in content quality and patient education. Health organisations must prioritize the production of approved educational videos and collaborate with institutions such as the Angioedema Centres of Reference and Excellence (ACARE) [46] and the European Academy of Allergy and Clinical Immunology (EAACI). Additionally, health care professionals must receive proper training or collaborate with content creators to ensure high-quality medical information. While patient-created videos offer valuable perspectives, they should also be vetted for accuracy. Enhancing the reliability of YouTube content will improve health literacy and provide better resources for both the public and medical professionals.

## Supplementary Information

Additional file 1.

## Data Availability

"The video link data for this study has been securely stored by our team, and access to the data can be provided upon request."
