# Supplementary material for: The origin of YouTube videos on hereditary angioedema matters
Source: Allergy Asthma Clin Immunol. 2025 Mar 19;21:12. doi: 10.1186/s13223-025-00947-6 (PMC11924627; doi:10.1186/s13223-025-00947-6)
Supplement: Supplementary file 1 — Additional file 1. [file 13223_2025_947_MOESM1_ESM.docx]

**Suppl Table 1: Scales used to evaluate the quality, reliability, understandability and actionability of the video content (27,33-35)**

| **Global Quality Score (GQS)** | |
| --- | --- |
|  |  |
| **1:** Poor quality | Poor flow of the video. Most information missing; not at all useful for patients |
| **2:** Generally poor quality and poor flow | Some information listed, but many important topics missing; of very limited use for patients |
| **3:** Moderate quality,  suboptimal flow | Some important information adequately discussed, but other information poorly discussed; somewhat useful for patients |
| **4:** Good quality and  generally good flow | Most of the relevant information listed, but some topics not covered; useful for patients |
| **5:** Excellent quality and flow | Very useful for patients |
| **PEMAT Tool for Audiovisual Materials (PEMAT-A/V)** | |
| **Understandability** |  |
| Content | |
| Item 1: | The material makes its purpose completely evident.  Disagree = 0       Agree = 1 |
| Word Choice and Style | |
| Item 3: | The material uses common, everyday language.  Disagree = 0       Agree = 1 |
| Item 4: | Medical terms are used only to familiarize the audience with the terms. When used, medical terms are defined.  Disagree = 0       Agree = 1 |
| Item 5: | The material uses the active voice.  Disagree = 0       Agree = 1 |
| Organization | |
| Item 8: | The material breaks or “chunks” information into short sections.  Disagree = 0       Agree = 1 Very short material=N/A |
| Item 9: | The material’s sections have informative headers.  Disagree = 0       Agree = 1 Very short material=N/A |
| Item 10: | The material presents information in a logical sequence.  Disagree = 0       Agree = 1 |
| Item 11: | The material provides a summary.  Disagree = 0       Agree = 1 Very short material=N/A |
| Layout and Design | |
| Item 12: | The material uses visual cues (e.g., arrows, boxes, bullets, bold, larger font, highlighting) to draw attention to key points.  Disagree = 0       Agree = 1 Video=N/A |
| Item 13: | Text on the screen is easy to read.  Disagree = 0       Agree = 1 No text or all text is narrated=N/A |
| Item 14: | The material allows the user to hear the words clearly.  Disagree = 0       Agree = 1 No narration=N/A |
| Use of Visual Aids | |
| Item 18: | The material uses illustrations and photographs that are clear and uncluttered.  Disagree = 0       Agree = 1 No visual aids=N/A |
| Item 19: | The material uses simple tables with short and clear row and column headings.  Disagree = 0       Agree = 1 No tables=N/A |
| **Actionability** | |
| Item 20: | The material clearly identifies at least one action the user can take.  Disagree = 0       Agree = 1 |
| Item 21: | The material addresses the user directly when describing actions.  Disagree = 0       Agree = 1 |
| Item 22: | The material breaks down any action into manageable, explicit steps.  Disagree = 0       Agree = 1 |
| Item 25: | The material explains how to use the charts, graphs, tables, or diagrams to take actions.  Disagree = 0       Agree = 1 No charts, graphs, tables, diagrams = N/A |
| **DISCERN** | |
| **Section 1** | |
| **Is the publication reliable?** | |
| 1. Are the aims clear?   No Partially Yes  1 2 3 4 5     1. Does it achieve its aims?   No Partially Yes  1 2 3 4 5     1. Is it relevant?   No Partially Yes  1 2 3 4 5     1. Is it clear what sources of information were used to compile the publication (other than the author or producer)?   No Partially Yes  1 2 3 4 5     1. Is it clear when the information used or reported in the publication was produced?   No Partially Yes  1 2 3 4 5     1. Is it balanced and unbiased?   No Partially Yes  1 2 3 4 5     1. Does it provide details of additional sources of support and information?   No Partially Yes  1 2 3 4 5     1. Does it refer to areas of uncertainty?   No Partially Yes  1 2 3 4 5 | |
| **Section 2** | |
| **How good is the quality of information on treatment choices?** | |
| 1. Does it describe how each treatment works?   No Partially Yes  1 2 3 4 5     1. Does it describe the benefits of each treatment?   No Partially Yes  1 2 3 4 5     1. Does it describe the risks of each treatment?   No Partially Yes  1 2 3 4 5     1. Does it describe what would happen if no treatment is used?   No Partially Yes  1 2 3 4 5     1. Does it describe how the treatment choices affect overall quality of life?   No Partially Yes  1 2 3 4 5     1. Is it clear that there may be more than one possible treatment choice?   No Partially Yes  1 2 3 4 5     1. Does it provide support for shared decision-making?   No Partially Yes  1 2 3 4 5 | |
| **Section 3** | |
| **Overall rating of the publication** | |
| 1. Based on the answers to all of the above questions, rate the overall quality of the publication as a source of information about treatment choices.   Low Moderate High  1 2 3 4 5 | |
| **Modified DISCERN** | |
|  | |
| 1. Are the aims clear and achieved?   No = 0       Yes = 1 | |
| 1. Are reliable sources of information used?   No = 0       Yes = 1 | |
| 1. Is the information presented balanced and unbiased?   No = 0       Yes = 1 | |
| 1. Are additional sources of information listed for patient reference?   No = 0       Yes = 1 | |
| 1. Are areas of uncertainty mentioned?   No = 0       Yes = 1 | |

**Abbreviations:** NS – Not significant. GQS – Global Quality Score (1-5). PEMAT-A/V – Patient Education Materials Assessment Tool for Audiovisual (0-100, higher scores indicate better understandability and actionability). DISCERN – A tool for assessing the quality of written health information on treatment choices (16-75, higher scores indicate better quality. Modified DISCERN – An adaptation of the DISCERN tool for audiovisual materials (0-5, higher scores indicate better quality).
